# Targeting therapy of hepatocellular carcinoma with doxorubicin prodrug PDOX increases anti-metastatic effect and reduces toxicity: a preclinical study

**DOI:** 10.1186/1479-5876-11-192

**Published:** 2013-08-21

**Authors:** Qun Wang, Yan-Jun Zhong, Jing-Ping Yuan, Li-Hua Shao, Jue Zhang, Li Tang, Shao-Ping Liu, Ya-Ping Hong, Raymond A Firestone, Yan Li

**Affiliations:** 1 Department of Oncology, Zhongnan Hospital of Wuhan University, Hubei Key Laboratory of Tumor Biological Behaviors & Hubei Cancer Clinical Study Center, No 169 Donghu Road, Wuchang District, 430071 Wuhan, P.R. China; 2 Princeton Global Synthesis LLC, 360 George Patterson Blvd. Suite 206, 19007 Bristol, PA, USA; 3 Nanjing Meihua Pharmaceuticals, Ltd, 210009 Nanjing, P.R. China

**Keywords:** Hepatocellular carcinoma, Molecular targeting therapy, Doxorubicin, PDOX, Metastases

## Abstract

**Background:**

This study was to investigate the effects and safety of cathepsin B-cleavable doxorubicin (DOX)-prodrug (PDOX) for targeting therapy of metastatic human hepatocellular carcinoma (HCC) using DOX as a positive control drug.

**Methods:**

The orthotopic nude mice model of highly metastatic HCC was established and the animals were randomized and treated with PDOX, DOX and saline, respectively. Hematology, biochemistry and tumor markers were studied. At autopsy, liver tumor weight and size, ascites, abdominal lymph nodes metastases, experimental peritoneal carcinomatosis index (ePCI), and tumor-host body weight ratio were investigated. Immunohistochemical studies and western blotting were done to investigate key molecules involved in the mechanism of action.

**Results:**

Compared with Control, both PDOX and DOX could similarly and significantly reduce liver tumor weight and tumor volume by over 40%, ePCI values, retroperitoneal lymph node metastases and lung metastases and serum AFP levels (*P <* 0.05). The PDOX group had significantly higher WBC than the DOX group (*P <* 0.05), and higher PLT than Control (*P <* 0.05). Serum BUN and Cr levels were lower in the PDOX group than DOX and Control groups (*P <* 0.05). Compared with Control, DOX increased CK and CK-MB; while PDOX decreased CK compared with DOX (*P <* 0.05). Multiple spotty degenerative changes of the myocardium were observed in DOX-treated mice, but not in the Control and PDOX groups. PDOX could significantly reduce the Ki-67 positive rate of tumor cells, compared with DOX and Control groups. PDOX produced the effects at least via the ERK pathway.

**Conclusion:**

Compared with DOX, PDOX may have better anti-metastatic efficacy and reduced side effects especially cardio-toxicities in this HCC model.

## Background

Hepatocellular carcinoma (HCC) is the fifth most frequent malignant tumors, and the third leading cause of cancer-related mortality in the world [1]. HCC patients are usually diagnosed when the tumor is in an advanced stage and lose the opportunity for curative surgery [2]. Other treatments including loco-regional or systemic chemotherapy, fail mainly due to the chemoresistance of tumor and inability to endure treatment responses [3].

One of the most commonly used chemotherapy drugs for HCC is doxorubicin (DOX), but high doses of DOX result in severe toxicities, such as hematological, gastrointestinal, renal, hepatic toxicities, and particularly cardiac toxicities [4-6].

Increasing evidence supports the role of cathepsin B (Cat B) in tumor invasion and metastasis [7-9], including HCC progression [10]. Cat B expression is increased in many cancers at the mRNA, protein and activity levels, and closely related to invasive behavior of cancer [11]. Therefore, Cat B could be a potential target for new drugs designed specifically against invading cancer cells.

To retain the therapeutic effect while reducing the toxicity of DOX, Dubowchik et al. [12-14] designed a smart prodrug of DOX, Ac-Phe-Lys-PABC-DOX (PDOX), in which a Cat B-specific dipeptide is introduced, along with a spacer PABC (*para*-aminobenzyloxycarbonyl) to increase the distance between dipeptide and DOX, so that the dipeptide can enter the Cat B’ active site. As a result of this molecular re-structuring, the prodrug is inactive in blood circulation and normal tissues where little Cat B exists in the active form. When the prodrug reaches Cat B-enriched area such as the invasion front of cancer, the Phe-Lys dipeptide is cleaved by Cat B, exposing the PABC spacer that is then hydrolyzed spontaneously, releasing free DOX at the cancer invasion front. Thus this prodrug could exert cytotoxicity to invading cancer cells while protecting normal cells from excessive drug exposure, a strategy called passive targeted therapy.

In our previous animal model study, we investigated the activities and side effects of PDOX to treat peritoneal carcinomatosis (PC) from gastric cancer, which suggests that PDOX might be a promising new drug against cancer invasion [15]. Inspired by the initial results, we designed this study to further explore the treatment potential of this prodrug in a more aggressive and highly lethal orthotopic nude mice model of HCC.

## Materials and methods

### Agents and drugs

The prodrug PDOX was synthesized according to the previously reported chemical process [12-14]. The molecular formula of PDOX is C_52_H_59_N_5_O_16_ · HCl, and the molecular weight is 1046.51. In terms of equivalent mole content, 1.8 mg PDOX is equivalent to 1 mg DOX (molecular weight 579.99). Doxorubicin for injection (DOX, Pharmacia, Milan, Italy, 10 mg per vial) was obtained commercially.

### HCC cell lines and animal models

Highly metastatic human HCC cell line HCCLM9 was used for animal model construction. This cell line was obtained by cloning culture, and 9 rounds of successive in vivo pulmonary metastases selections as described previously [16,17]. Cells were grown in RPMI 1640 medium (Mediatech, Manassas, VA) supplemented with 10% fetal bovine serum (FBS) and 1% penicillin/streptomycin (Gibco, Carlsbad, CA). The cells were cultured in a humidified atmosphere at 37°C in 5% CO_2_ and passaged if grown to 90% confluence.

### Orthotopic nude mice model of HCC and treatment

Male athymic BALB/c nu/nu mice, 4–6 weeks old, were obtained from Beijing HFK Bio-Technology Co. Ltd [animal quality certificate No. SCXK (jing) 290004] and housed in specific pathogen-free (SPF) condition at the Animal Experiment Center of Wuhan University. All animal experiments were carried out in accordance with the guidelines and approved protocols of the University of Wuhan Animal Experiment Center Institutional Animal Care and Use Committee (Permit Number 00024763).

**Figure 1:**
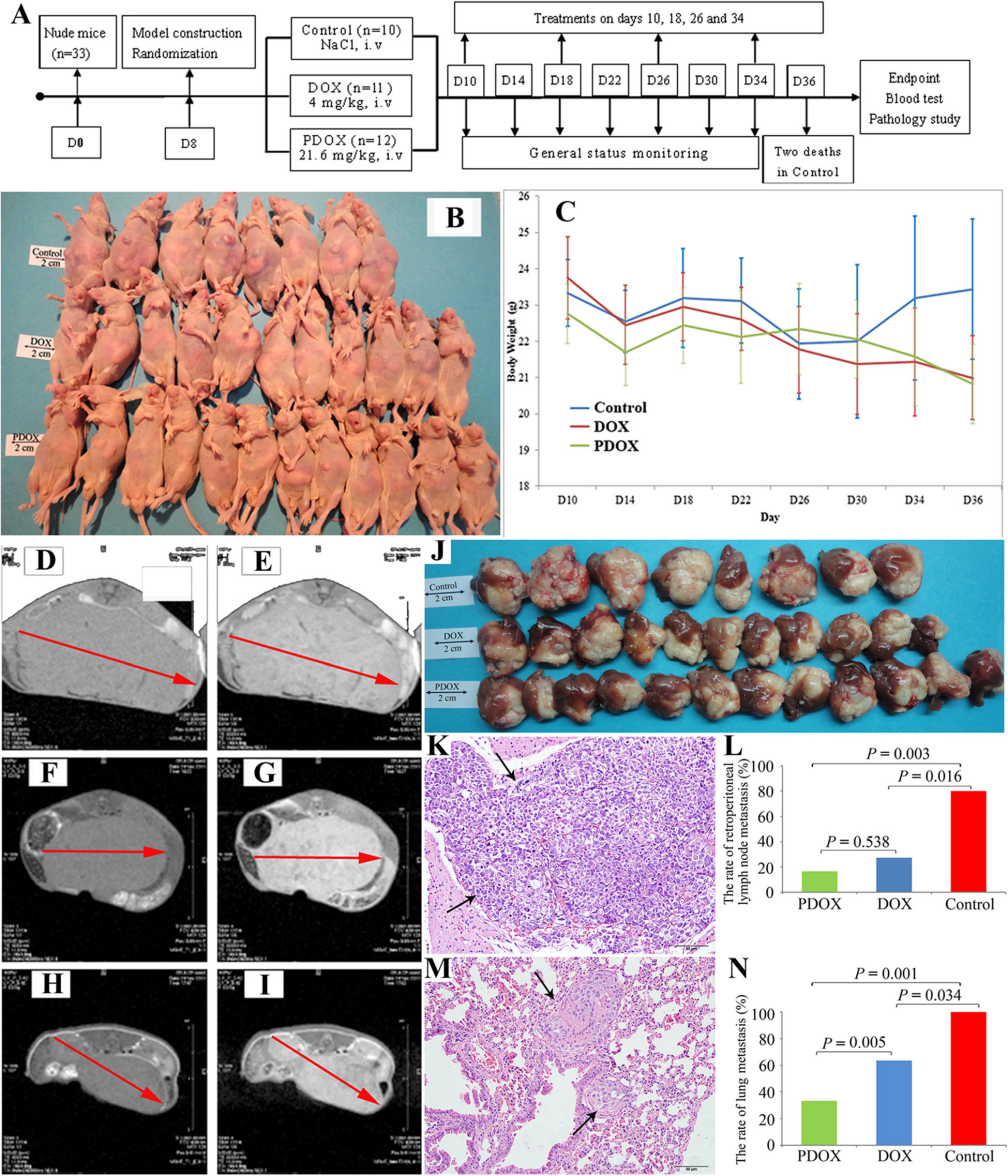
**The study procedure and major results. A**: The flow chart of this study. Nude mice were kept for 3 d of adaptation, and orthotopic model of human HCC was constructed as described in the Materials and Methods section. On d 8, the animals were randomized into 3 groups, and treated by tail vein injection of normal saline, DOX and PDOX, respectively. At the study endpoint, all animals were euthanized, and detailed pathological studies. **B**: The general status of animals appeared best in the PDOX, better in DOX, and worst in Control that were only 8 animals because 2 nude mice died before the study endpoint due to excessive tumor burden. **C**: The body weight curves at different time points show similar changes in the PDOX and DOX. In Control, there was a progressive increase on the last 6 d, due to accelerated tumor growth and increased ascites. **D**-**I**: Representative MRI scans of the liver tumors in Control **(D** for T1 and **E** for T2**)**, the DOX **(F** for T1 and **G** for T2**)** and the PDOX **(H** for T1 and **I** for T2**)** showed marked differences in tumor size. **J**: Both PDOX and DOX had similar and significant liver tumor growth inhibition compared with Control that 2 animals died on the morning of d 36, therefore only 8 tumors were available in the final picture presentation. **K** &**L**: PDOX and DOX treatments significantly reduced retroperitoneal lymph nodes metastases (arrows) compared with Control. **M** &**N**: The lung metastases (arrows) were also significantly reduced in the PDOX and DOX, and PDOX had greater inhibitory effect on lung metastases than. DOX. **D**: day; T1: T1-weighted image; T2: T2-weighted image; **K** &**M**: hematoxylin and eosin stain, 200×, scale bar = 50 μm.

Nude mice model with spontaneous pulmonary metastasis was established as described previously [17]. Briefly, HCCLM9 cells (5 × 10^6^ cells each) in 0.1 ml phosphate buffered saline (PBS) were injected subcutaneously into the left upper flank of 2 nude mice. The subcutaneous tumors were removed when they reached 8 mm in diameter, and minced into pieces (1 mm^3^) to perform orthotopic transplantation into livers of nude mice (n = 33). On day 8 after model establishment, the mice were randomized into Control group (n = 10), DOX group (n = 11), and PDOX group (n = 12), and treated as illustrated in the flow chart of Figure 1 (Figure 1A).

In our previous study [15], we found that by intraperitoneal injection, PDOX could be safely administered at twice the dose of DOX on a molar basis. Therefore, we increased the doses of both DOX (4 mg/kg) and PDOX (21.6 mg/kg) in this study, in which the PDOX dose was 3 times that of DOX on a molar basis.

The behaviors and general conditions were monitored daily, and body weights of animals were recorded twice a week. On d 36 post inoculation, the mice were anesthetized by peritoneal injection of 3% phenobarbital chloride, and then subjected to magnetic resonance image (MRI) study to measure the liver tumor size (Bruker Biospec 4.7 T/30, Germany). The peripheral blood was obtained for routine blood tests and biochemistry studies. Tumor tissues and major organs including the heart, the liver, the lungs and any other suspected organs were collected, fixed with 4% paraformaldehyde and embedded in paraffin for pathological and immunohistochemistry studies. In addition, fresh tumor tissues were obtained for western blotting studies as detailed below.

### Experimental peritoneal carcinomatosis index (ePCI)

An experimental peritoneal carcinomatosis index (ePCI) system was developed to evaluate the efficacy that took into consideration of tumor nodule sizes, distributions, and the characteristics of ascites. In this system, the abdominal cavity of the mouse was divided into 4 regions: region I, subdiaphragm; region II, the liver, spleen, stomach, and affiliated ligaments; region III, the small intestine, colon, mesenterium, and abdominal wall; and region IV, the pelvic cavity, urogenital system, and rectum. The detailed scoring criteria were modified from a similar reporting system on a rat peritoneal carcinomatosis model [18] and set forth in our previous report [15].

### Immunohistochemistry study

Tumor tissues obtained from animals of 3 groups were subjected to immunohistochemistry to detect the expressions of Cat B, Ki-67, CD34, VEGF, E-cadherin and D2-40, according to our previously reported procedures [15]. The primary antibodies for Cat B (Cat No 3190–100, BioVision, CA, USA, dilution 1:200), Ki-67 (MAB-0129, Maxim-Bio Co, CHN, Working solution), CD34 (BA0532, WuHan Boster Bio-Engineering Co, CHN, dilution 1:100), VEGF (RB-9031, Maxim-Bio Co, CHN, Working solution), E-cadherin (MAB-0589, Maxim-Bio Co, CHN, Working solution) and D2-40 (AM0103, Ascend Biotechnology Co, CHN, Working solution) were prepared and incubated with the slides for 2 h in a moist chamber. After a new cycle of washes, the slides were again placed in a moist chamber for 30-minute incubation with a biotinylated secondary antibody and biotin-peroxidase complex (Biogenex, SF, USA, Working solution). The color of immunoperoxidase reaction was achieved by immersion for 5 min in a solution containing the DAB chromogen (3,5-diamino-benzidine tetra-hydrochloride) and counterstained with hematoxylin for 2 min. The slides were observed under the microscope.

For the evaluation of IHC results, positive cells were stained brownish granules in the cell membrane, cytoplasm or nucleus. In all cases, cytoplasmic Cat B expression was scaled as moderate and strong expression. Ki-67 expressed in the nucleus. VEGF positive cells were stained both in the nucleus and cytoplasm. The expression of E-cadherin mainly existed in cell membrane and cytoplasm. CD34 and D2-40 positive cells were stained in cytoplasm. Ten fields in each slide were selected randomly and observed at a magnification of × 200. The expression of Ki-67 was evaluated according to positive rate. The positive expression of CD34 and D2-40 was evaluated according to microvessel density (MVD) and lymphatic microvessel density (LMVD).

### Western blotting study

Fresh tumor tissues in RIPA lysis buffer containing 1 μg/ml PMSF, 1 × Cocktail, were manually homogenized on ice using a glass homogenizer, then centrifuged at 3000 g for 10 min to remove cellular and nuclear debris. The protein concentration was determined using a BCA Assay kit (Biyuntian).

To determine the expressions of p-ERK1/2, ERK1/2, Bcl-2, caspase-3, and β-actin using western blotting, 100 μg total proteins were separated by SDS-polyacrylamide gel electrophoresis (4% stacking and 10% separating gels) and then transferred overnight onto PVDF membranes, which were blocked with 5% skimmed milk in 0.01 M phosphate buffer solution (PBS) containing 0.05% (v/v) Tween. Next, they were immunoblotted with a rabbit anti-human p-ERK (4370, CST, MA, USA, dilution 1:1000), rabbit anti- human ERK (4695, CST, MA, USA, dilution 1:1000), rabbit anti-human Bcl-2 (2870, CST, MA, USA, dilution 1:1000), rabbit anti-human caspase-3 (9665, CST, MA, USA, dilution 1:1000), mouse anti-human caspase-9 (9508, CST, MA, USA, dilution 1:1000), and rabbit anti-human β-actin (Santa Cruz, CA, USA, dilution 1:1000) for 3 h. Blots were then incubated with a peroxidase-conjugated sheep anti-rabbit IgG (Santa Cruz, CA, USA, dilution 1:8000) or sheep anti-mouse IgG (Santa Cruz, CA, USA, dilution 1:8000) for 2 h and developed using chemiluminescent detection with a Supersignal West Pico assay kit (Thermo, IL, USA) and autoradiography film.

### Blood tests and biochemistries

On d 36, animals were euthanized, and blood was obtained for routine studies, including peripheral blood profiles by Sysmex KX-21 automated hematology analyzer (Sysmex, Kobe, Japan); liver function parameters alanine aminotransferase (ALT), aspartate aminotransferase (AST), gamma-glutamyl transpeptidase (GGT), total bilirubin levels (TBIL), and direct bilirubin (DBIL) levels; renal function parameters blood urea nitrogen (BUN) and creatinine (Cr) levels; cardiac function parameters creatine kinase (CK), creatine kinase-MB (CK-MB) and lactate dehydrogenase (LDH) levels; electrolytes (K^+^, Na^+^, Ca^2+^, Mg^2+^ and Cl^-^) and serum alpha fetoprotein (AFP) levels; all by Aeroset Clinical Chemistry Analyzer (Abbott Laboratories, IL, USA).

### Statistical analysis

All data were analyzed using the statistical software of SPSS 13.0 for Windows (SPSS Inc, Chicago, USA). The differences in body weights, liver tumor weights, and the expression of Ki-67, CD34 and D2-40 among different groups were tested by one-way ANOVA. The differences of Cat B, VEGF and E-cadherin were analyzed by the chi-square (χ^2^) test. *P*-value < 0.05 was considered as statistically significant.

## Results

### PDOX had better effects on general status and similar inhibitory effects on liver tumor growth and loco-regional metastases

After tumor inoculation into the liver, the animals in the DOX and PDOX groups showed slight and progressive body weight decreases till the study endpoint. The general status of animals appeared better in the PDOX group than the DOX group, which in turn was better than Control group (Figure 1B). In the Control group, the animals showed body weight increases from d 30 to d 36, mainly due to excessive liver tumor and ascites (Figure 1C).

Prominent liver tumors were observed in all animals, and representative MRI abdominal scan of liver tumors were shown (Figure 1D to 1I**)**. At the study endpoint, the tumor weights were 6657.4 ± 1312.9 mg in the Control group, 3860.0 ± 1023.6 mg in the DOX group, and 3757.6 ± 603.5 mg in the PDOX group (*P* < 0.001, Control *vs* PDOX; *P* < 0.001, Control *vs* DOX; *P* > 0.05, DOX *vs* PDOX). Compared with Control, PDOX and DOX treatments reduced tumor weights by 43.6% and 42.0%, respectively. Similarly, PDOX and DOX treatments reduced tumor volumes by 53.4% and 49.1%, respectively (*P* < 0.01, DOX/PDOX *vs* Control) (Figure 1J, Table 1). The tumor-weight to body-weight ratio was also significantly reduced from 27.94% in the Control group to 18.28% in the DOX group and 18.10% in the PDOX group (*P* < 0.001, DOX/PDOX *vs* Control). The serum AFP level was reduced from 97.27 ± 34.22 ng/mL in the Control group to 24.69 ± 12.09 ng/mL in the DOX group and 22.31 ± 13.42 ng/mL in the PDOX group (*P* < 0.001, DOX/PDOX *vs* Control) (Table 1).

**Table 1:** Effects on tumor growth and metastases

**Items**	**Treatment groups**					***P *value**
	**Control (n = 10)**	**DOX (n = 11)**		**PDOX (n = 12)**		
		**Value**	**Inhibition ratio**^**☆**^	**Value**	**Inhibition ratio**^**☆**^	
**Liver tumor growth**						
Tumor weight (mg)	6657.4 ± 1312.9^*,§^	3860.0 ± 1023.6	42.0%	3757.6 ± 603.5	43.6%	^*^<0.001*vs* DOX ^§^**<0.001***vs* PDOX
Tumor volume (mm^3^)	4965.2 ± 2112.4^*,§^	2526.9 ± 1360.1	49.1%	2313.2 ± 675.7	53.4%	^*^<0.05*vs* DOX ^§^**<0.001***vs* PDOX
TW/BW ratio	27.94 ± 4.10^*,§^	18.28 ± 4.12	34.5%	18.10 ± 3.18	35.2%	^*^<0.001*vs* DOX ^§^**<0.001***vs* PDOX
AFP level (ng/mL)	97.27 ± 34.22^*,§^	24.69 ± 12.09	74.6%	22.31 ± 13.42	77.1%	^*^<0.001*vs* DOX ^§^**<0.001***vs* PDOX
ePCI	9 ± 2^*,§^	6 ± 2	33.3%	6 ± 2	33.3%	^*^<0.05*vs* DOX ^§^**<0.05***vs* PDOX
**Tumor metastases**						
Mediastinal LN meta	70.0% (7/10)	63.6% (7/11)	9.1%	33.3% (4/12)	52.4%	>0.05
Lung meta	100% (10/10) ^*,§^	63.6% (7/11)^#^	36.4%	33.3% (4/12)	66.7%	^*^<0.05*vs* DOX ^§^**<0.05***vs* PDOX ^**# **^**<0.05***vs* PDOX
Diaphragm meta	90% (9/10)	72.7% (8/11)	19.2%	50% (6/12)	44.4%	>0.05
Intrahepatic meta	30% (3/10)	27.3% (3/11)	9.0%	50% (6/12)	−66.7%	>0.05
Spleen ligament meta	70% (7/10)	36.4% (4/11)	48.0%	41.7% (5/12)	40.4%	>0.05
Heptogastric ligament meta	90% (9/10)	90.9% (10/11)	−1.0%	83.3% (10/12)	7.4%	>0.05
Renal ligament meta	40.0% (4/10)	36.4% (4/11)	9.0%	58.3% (7/12)	−45.8%	>0.05
Adrenal meta	10.0% (1/10)	0.0% (0/11)	100%	0.0% (0/12)	100%	>0.05
Mesenteric meta	90.0% (9/10)	81.8% (9/11)	9.1%	58.3% (7/12)	35.2%	>0.05
Retroperitoneal LN meta	80.0% (8/10) ^*,§^	27.3% (3/11)	65.9%	16.7% (2/12)	79.1%	^*^<0.05*vs* DOX ^§^**<0.05***vs* PDOX
Abdominal wall meta	80.0% (8/10)	45.5% (5/11)	43.1%	66.7% (8/12)	16.6%	>0.05
Bloody ascites	50.0% (5/10)	9.1% (1/11)	81.8%	25.0% (3/12)	50.0%	>0.05

*TW*: tumor weight; *BW*: body weight; *AFP*: alpha-fetoprotein; *ePCI*: experimental peritoneal carcinomatosis index; *LN*: lymph nodes; *Meta*: metastasis.

^**☆**^All the inhibition ratios were compared with the Control group. *,§, #: denote statistical significances for comparisons of the same item in different groups, as clearly described in the P value column.

In addition to liver tumor reduction, the loco-regional metastases were also investigated. We used the ePCI score system to evaluate the peritoneal metastases of this model. The ePCI was reduced from 9 ± 2 in the Control group to 6 ± 2 in the DOX group and 6 ± 2 in the PDOX group (*P* < 0.05, DOX/PDOX *vs* Control). Another significant effect was observed on retroperitoneal lymph node metastases, which occurred in 80.0% (8/10), 27.3% (3/11) and 16.7% (2/12) of animals, respectively, in the Control, DOX and PDOX groups (*P* < 0.05, DOX/PDOX *vs* Control) (Figure 1K and 1L, Table 1).

### PDOX had better inhibitory effects on lung metastases than DOX

Treatment effects on distant metastases were also studied. The rates of animals with lung metastases were reduced from 100.0% (10/10) in the Control group to 63.6% (7/11) in the DOX group and 33.3% (4/12) in the PDOX group (*P* < 0.05, Control *vs* DOX; *P* < 0.05, Control *vs* PDOX; *P* < 0.05, DOX *vs* PDOX) (Figure 1M and 1N, Table 1).

### PDOX had higher inhibitory effect on tumor proliferation than DOX

**Figure 2:**
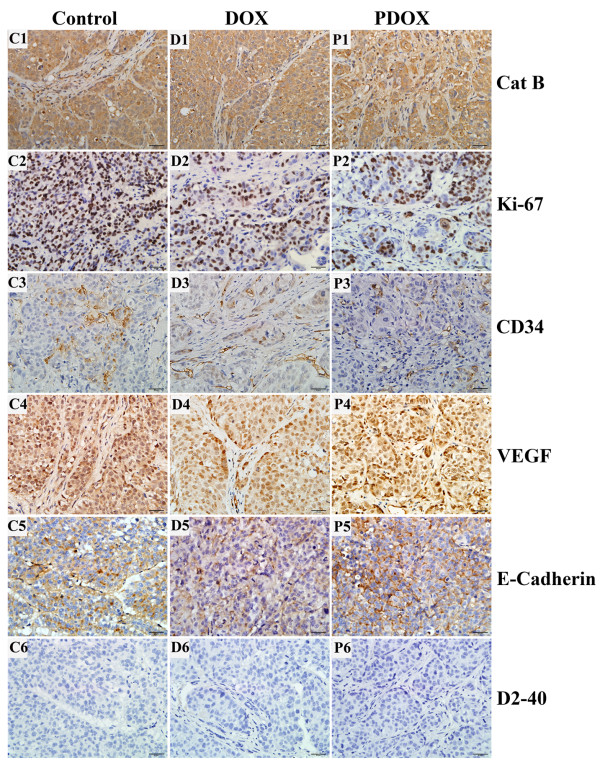
**Immunohistochemical studies on key molecules in tumor growth and metastases. C1, D1** &**P1**: the cytoplasmic expression of Cat B in 33 tumors was similar among the 3 groups. **C2**, **D2** &**P2**: The Ki-67 positive rate in the Control group **(C2**, 77.1%**)** was significantly higher than those in the DOX group **(D2**, 72.3%**)** and the PDOX group **(P2**, 61.6%**)** (Control vs DOX: *P* < 0.05, Control vs PDOX: *P* < 0.05, DOX vs PDOX: *P* < 0.05). **C3**, **D3** &**P3**: The CD34 positive micro-vessels density was similar among the 3 groups. **C4**, **D4** &**P4**: The rates of VEGF positive cells were similar among 3 groups. **C5**, **D5** &**P5**: The positive expression of E-Cadherin was similar among the 3 groups. **C6**, **D6** &**P6**: The D2-40 positive lymph vessels were also similar among the 3 groups. **C**: Control; **D**: DOX; **P**: PDOX. 400×, scale bar = 20 μm.

IHC studies were performed to investigate the expression of major cancer molecules possibly affected by the treatments. As shown in Table 2 and Figure 2, positive cytoplasmic Cat B expression was observed in all tumors from the 3 groups. Ki-67 positive rates were 77.1 ± 7.8% in the Control group, 72.3 ± 4.9% in the DOX group, and 61.6 ± 14.6% in the PDOX group (*P* > 0.05, Control *vs* DOX; *P* < 0.05, Control *vs* PDOX; *P* < 0.05, DOX vs PDOX). The median (range) MVD values of CD34 were 47.2 (21.4-70.0) in the Control group, 60.9 (37.0-91.2) in the DOX group, and 55.6 (22.2-80.2) in the PDOX group, respectively (*P* > 0.05). The VEGF positive rate was not statistically different among the 3 groups (*P* > 0.05). Similarly, there was no statistical difference in the expression of E-cadherin among the 3 groups (*P* > 0.05). The median (range) values of LMVD designated as D2-40 positive expression were 0.5 (0.0-3.2), 1.8 (0.0-8.4) and 1.8 (0.0-5.8) in the Control, DOX and PDOX groups, respectively (*P* > 0.05).

**Table 2:** Immunohistochemical analysis

**Items**	**Treatment groups**			***P *value**
	**Control (n = 10)**	**DOX (n = 11)**	**PDOX (n = 12)**	
Cat B	100% (10/10)	10% (11/11)	100% (12/12)	*P* > 0.05
VEGF	100% (10/10)	90.9% (10/11)	83.3% (10/12)	*P* > 0.05
E-cadherin	100% (10/10)	90.9% (10/11)	100% (12/12)	*P* > 0.05
Ki-67	77.1 ± 7.8%*	72.3 ± 4.9%^#^	61.6 ± 14.6%	*P < 0.05, Control *vs* PDOX ^#^*P* < 0.05, DOX vs PDOX
CD34	47.2 (21.4-70.0)	60.9 (37.0-91.2)	55.6 (22.2-80.2)	*P* > 0.05
D2-40	0.5 (0.0-3.2)	1.8 (0.0-8.4)	1.8 (0.0-5.8)	*P* > 0.05

Notes: Cat B, VEGF and E-cadherin were evaluated according to the percentage of positive expression (number of animals with positive expression ÷ number of all animals in the group × 100%). The expression of Ki-67 was evaluated according to positive rate in every animal. The positive expression of CD34 and D2-40 was evaluated according to microvessel density (MVD) and lymphatic microvessel density (LMVD).

*, #: denote statistical significances for comparisons of the same item in different groups, as clearly described in the P value column.

### PDOX had less hematological and biochemical toxicities than DOX

The hematological and non-hematological toxicities were studied (Table 3). In peripheral blood routine, the white blood cells levels in PDOX mice were higher than DOX mice (1.98-folds, *P* < 0.05). The platelet levels were higher in the PDOX group (1.67-folds, *P* < 0.05) and the DOX group (1.59-folds, *P* > 0.05) compared with Control. There were no differences in red blood cells and hemoglobin levels among the 3 groups.

**Table 3:** Routine blood tests and biochemistry (expressed as mean ± SD)

**Items**	**Treatment groups**			***P *value**
	**Control (n = 8)**^**☆**^	**DOX (n = 11)**	**PDOX (n = 12)**	
**Peripheral blood routine**				
RBC (×10^12^/L)	8.06 ± 3.50	9.59 ± 0.65	9.52 ± 1.47	> 0.05
HGB (g/L)	139.00 ± 9.82	141.18 ± 9.62	138.83 ±19.52	> 0.05
WBC (×10^9^/L)	3.25 ± 1.50	2.71 ± 1.00^*^	5.37 ± 3.31^*^	**< 0.05**
PLT (×10^9^/L)	385.67 ± 102.72^*, §^	611.36 ± 176.40	644.33 ± 293.34	^*^ **< 0.05***vs* DOX ^§^ **< 0.05***vs* PDOX
**Liver functions**				
AST (U/L)	489.01 ± 95.85^*^	338.42 ± 75.47^§^	444.64 ± 114.01	^*^ **< 0.05***vs* DOX ^§^ **< 0.05***vs* PDOX
ALT (U/L)	172.83 ± 52.26	219.56 ± 165.57	216.11 ± 153.50	> 0.05
TBIL (μmol/L)	3.51 ± 0.42	3.40 ± 0.50	3.59 ± 0.67	> 0.05
DBIL (μmol/L)	3.21 ± 0.10	3.17 ± 0.11	3.19 ± 0.16	> 0.05
GGT (U/L)	30.28 ± 10.65 ^*, §^	15.75 ± 6.13	18.43 ± 11.06	^*^ **< 0.05***vs* DOX ^§^ **< 0.05***vs* PDOX
**Renal functions**				
BUN (mmol/L)	15.83 ± 1.72^*, §^	11.99 ± 3.33 ^#^	9.29 ± 2.40	^*^ **< 0.05***vs* DOX ^§^ **< 0.001***vs* PDOX ^#^ **< 0.05***vs* PDOX
Cr (μmol/L)	41.73 ± 3.85^*^	43.37 ± 4.95^§^	34.76 ± 7.67	^*^ **< 0.05***vs* PDOX ^§^ **< 0.05***vs* PDOX
**Electrolytes**				
K^+^ (mmol/L)	6.56 ± 0.60	6.58 ± 0.36	6.81 ± 0.59	> 0.05
Na^+^ (mmol/L)	160.82 ± 4.04	158.53 ± 2.21	158.50 ± 2.54	> 0.05
Cl^-^ (mmol/L)	114.88 ± 1.86^*^	113.23 ± 2.66	112.42 ± 1.58^*^	**< 0.05**
Ca^2+^ (mmol/L)	2.25 ± 0.10^*^	2.31 ± 0.08^§^	2.38 ± 0.08	^*^ **< 0.05***vs* PDOX ^§^ **< 0.05***vs* PDOX
Mg^2+^ (mmol/L)	1.15 ± 0.09	1.23 ± 0.18	1.15 ± 0.34	> 0.05

^☆^ Note: Two nude mice died before the study endpoint, leaving only 8 mice available for blood test of routine and biochemistry parameters. *,§, #: denote statistical significances for comparisons of the same item in different groups, as clearly described in the P value column.

*WBC*: white blood cell; *NEUT*: neutrophil; *RBC*: red blood cell; *HGB*: hemoglobin; *PLT*: platelet; *LYM*: lymphocyte; *PLT*: platelet count; *ALT*: alanine transaminase; *AST*: aspartate aminotransferase; *TBIL*: total bilirubin; *DBIL*: direct bilirubin; *GGT*: γ-glutamyltransferase; *Cr*: creatinine; *BUN*: Blood urea nitrogen; *CK*: creatine kinase; *CK-MB*: creatine kinase-myoglobin; *LDH*: lactic dehydrogenase.

In terms of liver functions, compared with Control, DOX and PDOX caused significant reduction in GGT and AST levels (*P* < 0.05, DOX/PDOX *vs* Control) (Table 3). There were no statistically significant differences in AST, TBIL and DBIL levels among the 3 groups.

In terms of renal functions, compared with Control, both DOX and PDOX resulted in significant reduction in serum BUN levels (*P* < 0.001, Control *vs* DOX; *P* < 0.05, Control *vs* PDOX), and BUN levels in the PDOX group were also significantly lower than those in the DOX group (*P* < 0.05). Furthermore, the serum Cr levels in the PDOX group were much lower than those of the Control and DOX groups (*P* < 0.05, PDOX *vs* Control; *P* < 0.05, PDOX *vs* DOX) (Table 3).

Electrolytes results demonstrated that Cl^-^ was reduced in PDOX compared with Control group (*P* < 0.05); But Ca^2+^ was increased in PDOX compared with the Control and DOX groups (*P* < 0.05, PDOX *vs* Control; *P* < 0.05, PDOX *vs* DOX) (Table 3).

### PDOX had less cardio-toxicity than DOX

**Figure 3:**
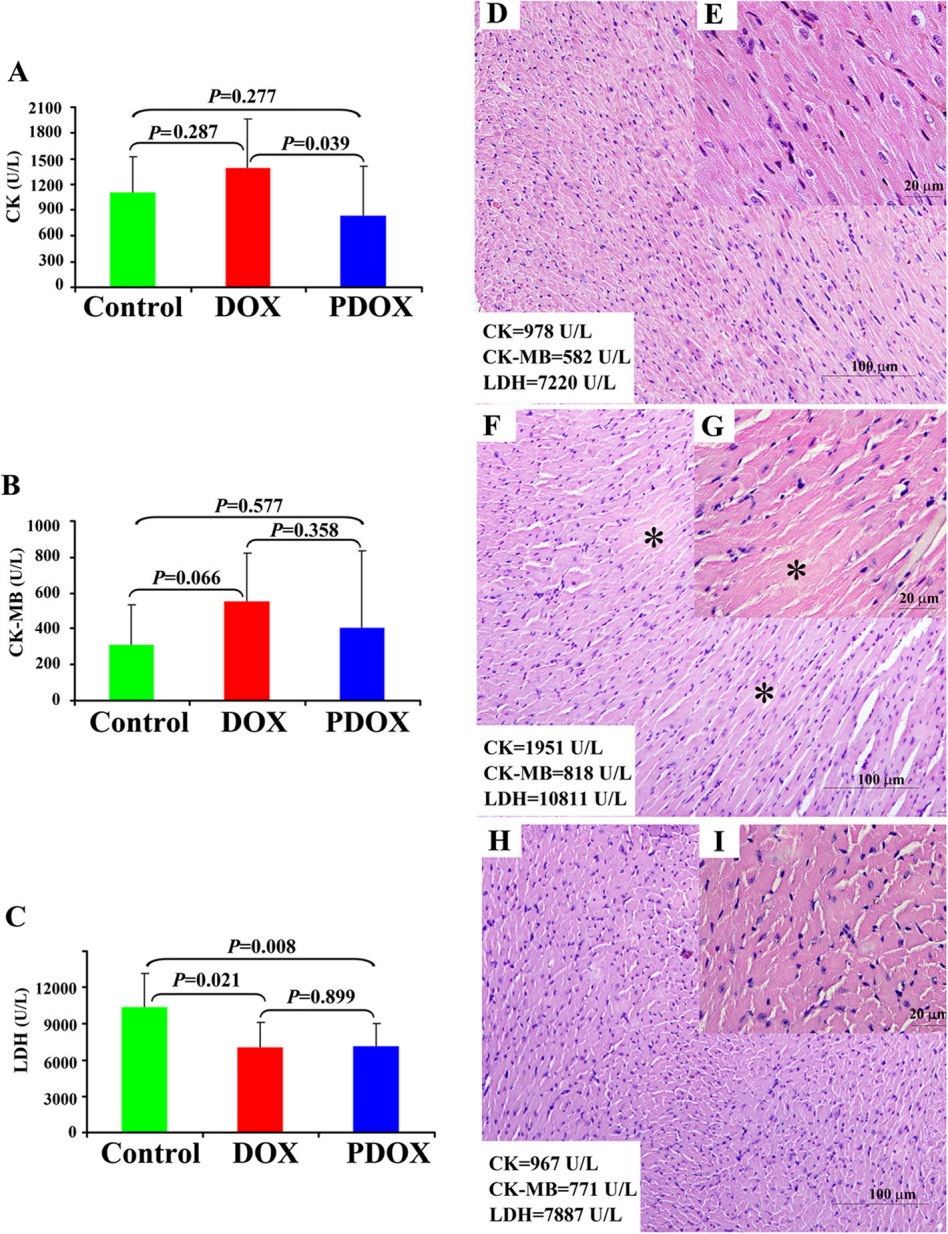
**The cardio-toxicities of animals in the 3 groups. A**: Compared with Control, DOX increased CK levels but without statistical significance, while PDOX significantly decreased CK levels compared with DOX (*P* < 0.05). **B**: Compared with Control, DOX increased CK-MB levels without significant difference, but PDOX did not increase CK-MB levels. **C**: Both DOX and PDOX significantly decreased LDH compared with Control group (*P* < 0.05 and *P* < 0.05). **D** &**E**: There were no observable histopathological changes in the myocardium of the Control mice. **F** &**G**: Multiple spotty degenerative changes were observed in the myocardium of the DOX-treated mice. **H** &**I**: There were no observable histopathological changes in the myocardium of the PDOX-treated mice.

Cardiac functions demonstrated that both DOX and PDOX significantly decreased LDH compared with Control group (*P* < 0.05, DOX/PDOX *vs* Control), but there were no differences between the DOX and PDOX groups. Compared with Control, DOX increased CK and CK-MB levels, although the differences didn’t reach the statistical significance. On the other hand, PDOX significantly decreased CK, compared with DOX (*P* < 0.05) (Figure 3A, 3B and 3C).

Histopathological study revealed multiple spotty degenerative changes in the myocardium in DOX-treated mice (Figure 3F and 3G). There were no observable histopathological changes in both Control and PDOX groups (Figure 3D, 3E, 3H and 3I).

### PDOX produced the effect at least by the ERK pathway

**Figure 4:**
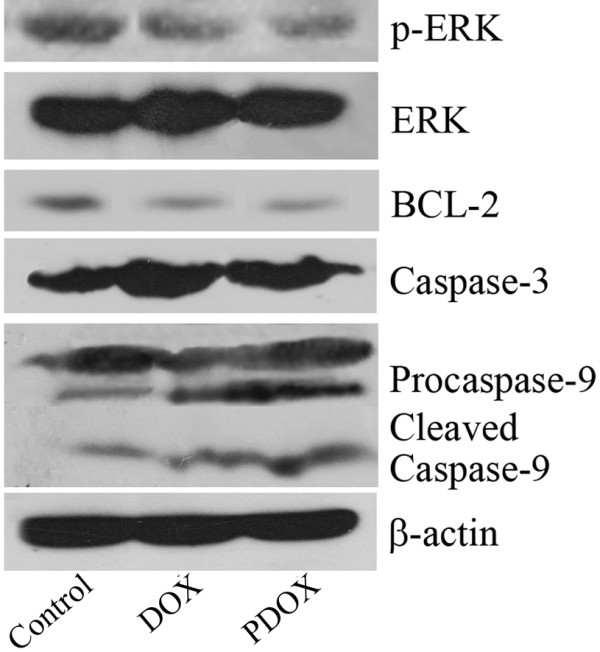
Western blotting showed that compared with Control, PDOX and DOX reduced ERK phosphorylation, decreased BCL-2 expression, and increased caspase-3 and caspase-9 activation.

To investigate the mechanism of PDOX producing effects, we used western blotting to study the expression of ERK, p-ERK, BCL-2, caspase-3, and caspase-9. The results showed that PDOX and DOX reduced ERK phosphorylation, decreased BCL-2 expression, and activated caspase-3 and caspase-9 (Figure 4).

## Discussion

Major treatment modalities for HCC are surgery, chemotherapy, regional therapies such as radiofrequency ablation, transarterial chemoembolization (TACE) and molecular targeting therapies. In either systemic chemotherapy or TACE, DOX is one of the most commonly used drugs with proven efficacy, but has serious side effects. Among 475 patients who received DOX in various studies, a 16% response rate was documented, with a median survival time of 3–4 months [19]. Significant grade 3 or above hematologic and gastrointestinal toxicities were encountered in patients treated with DOX, including neutropenia (63%), febrile neutropenia (17%), thrombocytopenia (24%), elevation of transaminases (13%), and diarrhea (7%) [20]. Therefore, it is imperative to modify DOX molecules in order to reduce its toxicities while maintaining its efficacy.

To fulfill this unmet clinical demand, Dubowchik et al. [12-14] designed a smart prodrug of DOX, which targets the active invading cancer cells and spares normal cells, because cancer cells, but not normal ones, secrete extracellular Cat B. It had previously been determined [12] that PDOX is stable in human and mouse blood. The major question, then, was whether PDOX reaches the tumor, is cleaved rapidly by Cat B, and the free DOX then enters into the cancer cells before it diffuses away. We now report, in this and in our previous paper [15], that PDOX indeed displays antitumor power at least equal to that of free DOX.

In this experimental study on a highly metastatic animal model of HCC, PDOX showed tumor inhibition similar to that of DOX, but significantly reduced toxicity profiles. Thus PDOX not only reaches the tumor as easily as free DOX, but is efficiently cleaved to free DOX there. In terms of liver tumor reduction, both PDOX and DOX treatments resulted over 40% of tumor growth inhibition. The general status of animals at the study endpoint also appeared better in the PDOX group. These results suggest that PDOX is at least as effective as DOX in this animal model.

Another possibility is that metastatic cells, which display more Cat B than those in the primary tumor [11], might have increased sensitivity to PDOX, and indeed they do. The primary tumor inhibition ratios relative to control by PDOX and DOX were 43.6% and 42.0%. In contrast, the metastases inhibition ratios relative to control by PDOX and DOX were 52.4% and 9.1% for mediastinal lymph nodes metastasis, 66.7% and 36.4% for lung metastasis, 44.4% and 19.2% for diaphragm metastasis, 35.2% and 9.1% for mesenteric metastasis, and 79.1% and 65.9% for retroperitoneal lymph nodes metastasis (Figure 1K and 1L, Table 1). As these are the principal sites of metastases of HCC, the superiority of PDOX over free DOX toward metastasis is remarkable and to our knowledge unprecedented, for usually metastases are more resistant than primaries to chemotherapy. It seems likely that the 3:1 molar excess of PDOX over DOX is more visible with metastasis than with the primary tumor because metastatic cells secrete more Cat B per cell than the primary.

In terms of toxicities, PDOX has shown advantages over DOX, even though the dosage of PDOX was 3 times that of DOX in this study. The peripheral blood cells counts such as WBC and PLT levels were significantly higher in the PDOX group than the DOX group, suggesting less bone marrow toxicity of PDOX. Moreover, serum BUN and Cr levels were also significantly lower in the PDOX group than the DOX group, suggesting less renal toxicity of PDOX. The most remarkable observation was the reduced cardio-toxicity in the PDOX group, compared with DOX, as demonstrated by CK, CK-MB, and LDH levels, and histopathological changes. Taking together, these facts support the notion that PDOX has increased anti-metastasis efficacy but reduced toxicities compared with DOX.

In order to explore the potential mechanisms of action of PDOX, we performed an IHC study and western blotting. Among the parameters investigated by IHC, we found that Cat B expression was strong in all tumors, providing supporting evidence that PDOX could produce the effect by this enzyme. Among other parameters related to tumor proliferation and invasion, Ki-67 reduction is the most prominent one in PDOX treated tumors. PDOX could reduce the Ki-67 positive rate by at least 15% compared with Control, and by at least 11% compared with DOX. The Ki-67 is expressed in all the other phases of the cell cycle except G0 phase, making it a reliable marker of active cell proliferation. High expression of Ki-67 has been linked with poor prognosis in prostate, breast, lung and hepatocellular carcinoma [21-24]. Therefore, significant reduction in Ki-67 positive rate could at least account for the fact that PDOX had better tumor inhibition than DOX in this study, although the difference between them did not reach statistical significance.

In addition to tumor proliferation parameters, tumor angiogenesis and lymphoangiogenesis were also studied. The expression of CD34 and VEGF positive endothelial cells may play an important role in understanding the process of angiogenesis in HCC and metastasis [25-27]. D2-40 and E-cadherin may provide important insights into the process of tumor-associated lymphangiogenesis [28-30]. In this study, the expressions of VEGF, CD34, D2-40 and E-cadherin were positive in all tumors, but there were no statistical differences among 3 groups. Therefore, we speculate that PDOX did not have different effects on tumor angiogenesis, lymphangiogenesis and cell adhesion.

The extracellular signal-regulated kinase (ERK) signaling pathway plays an important role in tumor invasion and metastasis [31,32]. Our study demonstrated that DOX and PDOX reduced ERK phosphorylation and BCL-2, activated casepase-3 and caspase-9, suggesting that PDOX produced the effect at least via ERK pathway.

Presently, knowledge regarding the biological processes of hepatocarcinogenesis has expanded significantly allowing the identification of the molecular processes involved in HCC development. Among these molecules, growth factors and neoangiogenesis factors with their receptors, tyrosine kinase intracellular enzymatic pathways and intracellular signal transmission factors have been under intensive study [33]. These substances represent potential molecular targets for targeted therapies with highly specific small molecules such as sorafenib, sunitinib, brivanib, cetuximab, erlotinib and lapatinib, which have emerged as promising therapeutic approaches for advanced HCC [34,35]. Many other molecular targeting agents to block epidermal growth factor receptor (EGFR), vascular endothelial growth factor receptor (VEGF), platelet-derived growth factor receptor (PDGFR), and mammalian target of rapamycin (mTOR) are also at different stages of clinical development for the treatment of advanced HCC [36-38].

The most successful drug of this kind is sorafenib, an orally-active multikinase inhibitor targeting both tumor cells and the tumor vasculature. It is the first agent to improve the overall survival of patients with advanced HCC, has been approved for molecular targeted therapy for patients with advanced HCC [39], representing a landmark success in the treatment of advanced HCC [40], even though the survival benefit of sorafenib is about 3 months for HCC patients with Child-Pugh Class A liver function, and less infrequent side effects such as hand-foot skin reaction (HFSR) [41,42].

Compared with these small molecules, PDOX could be termed as a “passive targeting agent”, which exerts its effect by Cat B cleavage. Normal organs are protected by masking the cytotoxic drug DOX with a simple dipeptide that renders it nontoxic. At the tumor the mask is removed by Cat B, a ubiquitous proteolytic enzyme that is so destructive to tissue that normally it occurs only within cells, encased in lysosomes. Only tumor cells secrete Cat B externally, confined to their plasma membranes, for the purpose of penetrating basement membrane and extracellular barriers during cancer invasion. The prodrug PDOX is rapidly cleaved by Cat B at the Phe-Lys bond. The resulting PABC-DOX decomposes at once to para-aminobenzyl alcohol, CO_2_ and free DOX. Furthermore, PDOX kills metastatic cancer cells (which are normally harder to kill than primary tumor cells) more powerfully than free DOX itself.

In summary, this study has provided more supporting evidence to show that PDOX does have increased anti-metastatic effects and reduced side effects especially the cardio-toxicity in this highly metastatic HCC model system. PDOX could be a promising new drug candidate for molecular targeting therapy of HCC.

## Abbreviations

HCC: Hepatocellular carcinoma; DOX: Doxorubicin; Cat B: Cathepsin B; PDOX: Ac-Phe-Lys-PABC-DOX; PC: Peritoneal carcinomatosis; FBS: Fetal bovine serum; SPF: Specific pathogen-free; PBS: Phosphate buffered saline; MRI: Magnetic resonance image; ePCI: Experimental peritoneal carcinomatosis index; MVD: Microvessel density; LMVD: Lymphatic microvessel density; ALT: Alanine aminotransferase; AST: Aspartate aminotransferase; GGT: Gamma-glutamyl transpeptidase; TBIL: Total bilirubin levels; DBIL: Direct bilirubin; BUN: Blood urea nitrogen; Cr: Creatinine; CK: Creatine kinase; CK-MB: Creatine kinase-MB; LDH: Lactate dehydrogenase; AFP: Alpha fetoprotein; TACE: Transarterial chemoembolization; EGFR: Epidermal growth factor receptor; VEGF: Vascular endothelial growth factor receptor; PDGFR: Platelet-derived growth factor receptor; mTOR: Mammalian target of rapamycin; HFSR: Hand-foot skin reaction.

## Competing interests

No potential conflicts of interest were disclosed.

## Authors’ contributions

WQ carried out the animal study, the gross pathology and biochemistry study, statistical analysis, and wrote the paper draft. ZYJ carried out the western blotting study and wrote part of the paper draft. YJP carried out the immunohistochemistry study. SLH, ZJ and TL help the animal study and part of the data analysis. LSP help biochemistry study. HYP and FR produced and provided the prodrug. LY designed, supervised and entire study process, and finalized the draft. All authors have read and approved the final manuscript.
